# Integrating Generative AI in Dental Education: A Scoping Review of Current Practices and Recommendations

**DOI:** 10.1111/eje.13074

**Published:** 2025-01-31

**Authors:** Sergio E. Uribe, Ilze Maldupa, Falk Schwendicke

**Affiliations:** ^1^ Department of Conservative Dentistry and Oral Health Riga Stradins University Riga Latvia; ^2^ Baltic Biomaterials Centre of Excellence Headquarters at Riga Technical University, Riga, Latvia & Institute of Stomatology, Riga Stradins University Riga Latvia; ^3^ Clinic for Conservative Dentistry and Periodontology LMU Klinikum Munich Germany

**Keywords:** artificial intelligence, dental education, generative artificial intelligence, implementation guidelines

## Abstract

**Background:**

Generative AI (GenAI) tools like ChatGPT are increasingly relevant in dental education, offering potential enhancements in personalised learning and clinical reasoning. However, specific guidance from dental institutions remains unexplored.

**Aim:**

To identify, analyse and summarise existing guidelines from universities and organisations on using GenAI in dental education, focusing on recommendations for academic staff.

**Methods:**

A scoping review (10.17605/OSF.IO/3XMP7) searched for GenAI guidance on university websites, search engines (Google Search, Scholar, Perplexity and PubMed) and through contacting relevant academics (January 2022 to June 2024). Two reviewers independently screened and extracted data, including implementation details, AI tools and permitted/prohibited uses. Thematic analysis revealed common applications, benefits, challenges and recommendations.

**Results:**

Thirty‐one unique documents were included from 21 universities in 15 countries and three international organisations. Thematic analysis identified common applications, benefits, challenges and recommendations for integrating GenAI, including facilitating teaching and learning, personalised learning, efficient content creation and encouraging critical thinking. However, challenges such as academic integrity, ethical use, bias and privacy issues were also identified. No dental education‐specific guidelines were found.

**Conclusion:**

This review identified and summarised existing GenAI guidelines from universities and organisations relevant to dental education. The guidelines emphasise ethical use, transparency, academic integrity, secure environments and AI misuse detection tools. However, the absence of dental specific guidance presents an opportunity to fill this gap, providing recommendations for academic staff to integrate GenAI effectively while promoting critical thinking and responsible AI use.

## 1 Introduction

Artificial intelligence (AI) is changing dental education by providing tools that enhance diagnostic accuracy, facilitate treatment planning and support the development of clinical decision‐making skills [1]. AI includes a broad spectrum of models capable of processing and analysing information to aid decision‐making and content creation [2]. Within AI, two main categories exist, traditional (or discriminative) AI and generative AI, each with distinct roles in dental education. Traditional AI, known for categorising data and identifying patterns, can analyse, for instance, dental images to distinguish between healthy and pathological tissues [1]. This type of AI operates within a defined task, such as identifying the presence of caries in dental images, and is particularly valuable for its accuracy in repetitive, structured scenarios. On the other hand, generative AI (GenAI) creates new data or content based on patterns learned from large datasets. A specialised form of generative AI, known as Large Language Models (LLMs), such as OpenAI's ChatGPT, offers transformative potential in dental education [3]. LLMs go beyond categorisation, allowing interactive learning experiences by simulating patient interactions, providing personalised feedback and generating detailed explanations or case scenarios [4]. These tools can enhance clinical reasoning and adapt learning content to individual student needs [5]. However, integrating GenAI requires managing challenges related to data privacy, content biases and the risk of academic misconduct, such as plagiarism [6]. Additionally, generative AI may produce inaccurate or misleading information, a phenomenon known as ‘hallucinations’, [7] which must be carefully addressed to ensure ethical and effective use in education.

While GenAI holds great promise for enhancing dental education and research, a recent global survey revealed a significant concern among educators: the need for clear guidelines or recommendations for the effective use of GenAI in dental education [3]. 2023 UNESCO global survey recently found that less than 10% of over 450 schools and universities have developed policies or formal guidance on GenAI applications [8]. This lack of formal guidance underscores the need for comprehensive frameworks to support safe, equitable and effective GenAI integration in education. Unlike other technologies in dental education, which primarily assist in diagnostics or imaging, GenAI directly influences learning interactions and content creation, warranting specific oversight [3].

This scoping review aims to identify, examine and analyse existing guidelines universities and organisations provide on using GenAI in education, primarily targeted at academic staff. The review aimed to extract, tabulate and identify common areas and recommendations, providing an overview and proposing minimal essential components for developing a guideline or policy on using GenAI in dental education.

## 2 Materials and Methods

### 2.1 Study Design, Protocol and Registration

This scoping review followed the methodology outlined by the Joanna Briggs Institute (JBI) guidelines for scoping reviews [9]. A review protocol was registered with the Open Science Framework (OSF) and is accessible at http://doi.org/10.17605/OSF.IO/3XMP7. No major deviations from the protocol were made during this review.

### 2.2 Eligibility Criteria

The documents were included based on the following criteria: official guidelines from universities with dental schools or faculties, a focus on GenAI in dental education, intended for academic staff, students, or both, no language restriction, and published between January 2022 and June 2024 (post‐ChatGPT release).

### 2.3 Information Sources

We emailed 437 academic contacts identified from a previous research study [3]. Each contact received up to three follow‐up emails asking whether their university had implemented any GenAI guidelines and, if so, requesting a copy or link to the document. We received 57 responses: 51 reported no existing guidelines, three provided documents or links to guidelines and three confirmed the presence of guidelines but could not share them. We searched the websites of top dental schools, as identified by QS rankings. Additionally, we utilised search engines, including Google Search, Google Scholar and Perplexity, to locate relevant guidelines. To broaden our reach, we also shared posts on social media platforms, inviting contributions and recommendations for GenAI‐related guidelines. One researcher conducted the searches.

### 2.4 Search

The search strategy for general search engines involved iterating the following terms: Artificial intelligence OR Generative artificial intelligence OR ChatGPT OR Large language models, Academic staff, lecturers, instructors, faculty members, professors, students, Dental Education OR Dentistry, Guidelines OR Policy OR Guidance OR Recommendations OR Implementation. These terms will be combined using Boolean operators (AND, OR) to ensure a comprehensive search. The search strings will be adjusted according to the search engine's requirements. The full details are available in Table S1.

### 2.5 Selection of Sources of Evidence and Inclusion Criteria

The process consisted of two phases. In the first phase, Title and Abstract Screening, two independent reviewers assessed the relevance of titles and abstracts, resolving discrepancies by consensus. In the second phase, the same reviewers conducted a full‐text review of guidelines that passed the initial screening, documenting reasons for exclusion. Inclusion criteria were AI in education guidelines from universities with dental schools, dental education associations and international higher education organisations. The most comprehensive one was included when multiple guidelines from the same organisation were found. Only guidelines for academic staff or both academic staff and students were considered; those exclusively for students were excluded as the focus was on implementation by academic staff.

### 2.6 Data Charting Process

The review team developed and piloted a standardised data extraction form. Two researchers extracted the data in duplicate, and consensus resolved disagreements. Data extraction included the following items:

- URL
- Publication date
- Paper title
- Authors (if none, the institution was listed)
- Institution
- Country (if applicable)
- Aim of the document
- Classification (guideline, policy, recommendation, other):
  ○ *Guideline*: A structured set of general principles or recommendations to guide decisions and actions without being prescriptive.
  ○ *Policy*: A formal, established protocol or standard adopted by an institution that is mandatory and enforceable, describing specific requirements and procedures.
  ○ *Recommendation*: An advisory statement suggesting a course of action, often based on evidence or best practices, which is not binding but influences decision‐making.
- Method of development (consensus, unclear or unspecified, other)
- Intended audience (academic staff, students, or both)
- Definitions presented (GenAI, what AI is, etc.)
- Mention of AI tools
- Permitted uses of GenAI
- Prohibited uses of GenAI
- Use cases provided
- Mentions of penalties or sanctions for inappropriate use

### 2.7 Critical Appraisal of Individual Sources of Evidence

In this scoping review, we described the individual sources of evidence without conducting a quality assessment. Our focus was on mapping the existing guidance and the contents of such guidance related to the use of GenAI in higher institutions with dental schools rather than evaluating the methodological quality of each source.

### 2.8 Synthesis of Results

Data synthesis involved a descriptive analysis of the study characteristics and findings. The unit of analysis was the document. The results were categorised thematically to identify common applications, benefits, challenges and recommendations for integrating GenAI into dental education. A narrative summary was provided to highlight key insights and gaps in the literature. Quantitative data, where applicable, were presented using summary statistics.

## 3 Results

The search identified 46 potential hits: 40 universities, five dental education organisations and one global education organisation (UNESCO). From the universities, 30 of 40 (75%) were identified through QS Rankings. Google Search and colleague recommendations accounted for five universities each (12.5%). Institutions may have multiple documents. From the organisations, all six were retrieved by Google search. After manual screening, 13 documents were excluded. Exclusions were due to duplication (five instances) and eight solely for students, which needed to meet the review's focus on guidelines for both students and academic staff (Table S2)—the final dataset comprised 31 unique documents from 21 universities and three associations. The geographic distribution included Africa with three documents from two countries (Egypt and South Africa) and one document from the Southern African Regional Universities Association (SARUA); Asia with two documents from two countries (China and Japan); Europe with the highest number of documents (10) from six countries (Belgium, Cyprus, Latvia, Sweden, Switzerland and the UK), plus one document from the European University Association (EUA); North America with eight documents from two countries (USA and Mexico); South America with one document from Chile; Oceania with two documents from two countries (Australia and New Zealand); and one document from a global organisation, the United Nations Educational, Scientific and Cultural Organization (UNESCO).

The analysis of documents from universities across various continents revealed several common themes and specific focus areas related to integrating GenAI into education. The ethical use of AI was a prominent theme, highlighted in 11 of 31 documents. These guidelines emphasised the importance of using AI responsibly to maintain academic integrity. Institutions such as the University of Michigan, Harvard University and the University of Zurich underscored the necessity of ethical AI to enhance teaching and learning. Transparency was frequently mentioned, with 9 out of 31 documents stressing the need for clear communication about AI usage to maintain accountability. Guidelines from the University of Sydney and Pontificia Universidad Católica de Chile (PUC) particularly emphasised this aspect.

Maintaining academic integrity was another key focus in 13 out of 31 documents. Universities like Duke University, the University of Washington and the University of Birmingham provided comprehensive policies to prevent academic dishonesty related to AI use, emphasising the need for proper supervision and documentation. Most documents, such as KU Leuven and Queen Mary University of London, advocated for the responsible use of AI tools. All the documents stressed the importance of clear communication on AI usage and fair assessment of student work. We found some guidance with specific use cases and detailed guidance on AI usage, supervision and documentation were common. For example, the University of Michigan, Riga Stradins University (RSU) and the University of California—San Francisco (UCSF) included specific tools like ChatGPT and DALL‐E, detailing their integration into teaching and assignments.

ChatGPT was the most frequently cited tool (13/31 documents), with institutions like Tokyo University, the University of Michigan, RSU and the University of Otago mentioning its use in teaching, learning and assessment. DALL‐E (5 documents), Microsoft Copilot (University of Sydney, University of Michigan), DeepL and Bing Chat (Tokyo University) were also mentioned for content creation, educational activities, administrative tasks and language translation. Other tools, such as Stable Diffusion, Gemini, Duet AI, Microsoft 365 Copilot, Bedrock and CodeWhisperer, were mentioned in the University of Michigan and the RSU document, demonstrating diverse AI technology use and a preference for ChatGPT.

Fifteen of 30 documents explicitly defined permitted and prohibited uses of AI tools. Examples include the University of Otago and the University of North Carolina, which allow AI to enhance learning and teaching but prohibit unauthorised use in assessments, and the University of Pretoria and Cairo University, which permit AI for educational purposes but restrict its use in scholarly work without proper oversight. Most penalties for unauthorised AI tool use targeted students rather than academic staff, focusing on academic misconduct like using AI in coursework, assessments or as original work. Only a few documents specified penalties for students and academic staff, primarily focusing on maintaining ethical standards and academic integrity. These institutions include the University of Otago, the University of Pretoria and the University of Michigan.

Some universities had unique elements in their guidelines. Tokyo University provided in‐depth overviews of AI limitations and ethical considerations specific to their academic contexts. The University of Michigan and UCSF School of Pharmacy specified tools such as ChatGPT and DALL‐E, highlighting their use in classroom integration and assignment design. The University of the Witwatersrand focused on promoting innovative teaching methods using AI while maintaining academic integrity. The PUC emphasised the importance of critical thinking, ethical use, and equitable access to AI tools. NYU and the University of Pretoria offered practical advice on safely and ethically using AI tools, including privacy considerations and understanding AI biases. Overall, the University of Michigan's guidelines were the most comprehensive, covering various AI technologies, classroom integration, assignment design, ethical considerations and clear permitted and prohibited uses, with penalties for unauthorised use. Riga Stradins University (53 pages) and UNAM (32 pages) had the longest guidelines among the universities.

The analysis of documents from the European University Association, SARUA and UNESCO revealed common themes and focus areas related to the responsible use of GenAI in higher education. Ethical use and responsible engagement with AI were prominent in all documents. The European University Association emphasised adapting learning and teaching approaches while maintaining ethical standards, similar to guidelines from universities like the University of Michigan and Harvard University.

Transparency was another key theme, especially in the UNESCO document, which focused on integrating AI into learning processes, research and administration while preventing misuse and biased information. This mirrors university guidelines from institutions like the University of Sydney and PUC, which stressed clear communication about AI usage. Innovation in teaching and learning was encouraged by all three organisations, with SARUA and the European University Association advocating for adapting teaching approaches and fostering critical thinking in students, comparable to the innovative methods promoted by universities like the University of the Witwatersrand.

As in the university documents, ChatGPT was the most frequently mentioned tool in all three documents. SARUA additionally mentioned GPTZero, which focuses on detecting AI‐generated content and UNESCO included DALL‐E, which is used for generating images from text descriptions. The European University Association and SARUA discussed using AI for efficiency, personalised learning and fostering innovation while warning against misuse leading to bias and privacy issues.

Each organisation's guidelines contained unique elements. The European University Association provided comprehensive guidance on adapting learning and teaching methods, SARUA emphasised critical engagement and innovation, and UNESCO offered a quick start guide to using ChatGPT in higher education, focusing on ethical considerations and the rapid development of AI technologies. These findings highlight the global recognition of AI's potential in higher education and underscore the importance of ethical and responsible integration to enhance learning, teaching and administrative processes.

Among the organisations, UNESCO's 48‐page Recommendation was the most comprehensive. It addressed GenAI challenges in education and research, focusing on security, privacy, copyright and manipulation concerns. UNESCO emphasised a human‐centred approach for ethical, safe, equitable, and meaningful use, recommending testing AI use cases, reviewing social and ethical implications, analysing environmental costs, making system‐wide adjustments, establishing GenAI effectiveness criteria and providing timely regulatory advice. The document highlighted the importance of addressing GenAI security and privacy issues, promoting human skills and collective action, and fostering inclusion, equity and cultural diversity. It offered comprehensive and actionable recommendations for policymakers and educational institutions. The full details of the documents from the universities and organisations are provided in Table 1.

**TABLE 1:** Summary of guidance from universities and organisations on GenAI in education.

Year	Organisation type	Document title	Institution	Country or region	Aim of the document	Type^a^	Development^b^	Target audience^c^	Mention tools	Present permitted uses of Gen AI	Prohibited uses of Gen AI^d^	Provide example cases (Y/N)	Mention penalties or sanctions (Y/N)	Focus on	Comments
2023	Association	SARUA: Southern African Regional Universities Association statement on ChatGPT and other AI tools	Southern African Regional Universities Association (SARUA)	Africa	Discuss the implications of ChatGPT and other AI tools in education	GL	ND	B	ChatGPT, GPTZero	Positive and critical engagement, innovation in learning and teaching	—	N	N	Engage with AI tools positively and critically, adapt learning, teaching and assessment approaches, foster innovation and critical thinking in students	Emphasises the need for universities to adapt and engage with AI development
2024	University	Frequently asked questions about generative AI at Sydney	University of Sydney	Australia	FAQs on generative AI in teaching	REC	ND	B	ChatGPT, Adobe Firefly, Microsoft Copilot	Learning and teaching	UA	Y	Y	Transparency, ethical use, student engagement	Practical tips and guardrails
2023	University	Responsible use of Generative Artificial Intelligence—KU Leuven Learning Lab	KU Leuven	Belgium	Responsible use of GenAI in education	REC	Unclear	A	—	—	—	N	N	Use GenAI responsibly, clear communication on AI usage, fair and correct assessment of student work	Emphasises responsible usage without banning
2024	University	Responsible use of Generative Artificial Intelligence—Student	KU Leuven	Belgium	Educate students on responsible use of GenAI	GL	ND	S	—	—	—	N	N	Engage with GenAI long‐term, maintain academic standards, understand and transparently use GenAI	Focuses on long‐term engagement and transparency
2024	University	Marco para el Uso de Inteligencia Artificial en Docencia	Pontificia Universidad Católica de Chile (PUC)	Chile	Promote responsible and ethical use of AI in teaching to enhance educational experiences while considering ethical implications and ensuring equitable access.	GL	ND	B	—	Ethical use in teaching, critical thinking development, support for equitable access	—	N	N	Emphasise the ethical use of AI, promote critical thinking and transparency, ensure equitable access to AI tools for all students	Reflects on the importance of ethical considerations and equitable access in the integration of AI in teaching
2024	University	Policy for AI in Education	The University of Hong Kong	China	Provide a framework for AI policy in education	GL	ND	B	—	Yes	Y	N	N	Importance of equal access to AI, need for security in AI usage, structured approach to implementing AI policy	
2023	University	Recommendations for the use of Artificial Intelligence in teaching processes at UCY	University of Cyprus	Cyprus	Provide recommendations for AI use in teaching	GL	ND	B	—	Enhance learning and teaching	VC	N	Y	Transparency, ethical use, critical thinking	Focus on transparency and ethical use
2023	University	FCAI‐GAI Use Guidelines	Cairo University	Egypt	Develop guidelines for using generative AI in teaching and research	GL	ND	B	ChatGPT, DALL‐E, Stable Diffusion	Use in coursework, projects, research proposals and thesis/dissertations	UA	Y	Y	Ethical use, transparency, academic integrity	Emphasises responsible usage without banning
2023	Association	Artificial intelligence tools and their responsible use in higher education learning and teaching	European University Association (EUA)	Europe	Guide responsible AI use in higher education	GL	Consensus	B	—	Use for efficiency, personalised learning	MI	N	N	Adapt learning and teaching approaches, responsible and ethical use of AI, embrace AI for innovation in education	Reflects on dynamic nature of AI in education
2023	Global organisation	ChatGPT and Artificial Intelligence in Higher Education: Quick Start Guide	UNESCO	Global	Introduce ChatGPT and its applications in higher education	GL	ND	B	ChatGPT, DALL‐E	Learning and administrative tasks	MI	Y	N	Embrace AI's potential in education, address ethical challenges, promote informed and critical use	Highlights rapid AI development and ethical considerations. 39 pages.
2023	University	Guidelines for Instructors Regarding AI in University Education	Tokyo University	Japan	Guide the use in education	REC	ND	A	ChatGPT, DeepL, Bing Chat	Enhance teaching and learning	UA	N	N	Understanding AI limitations, ethical considerations, academic integrity	Detailed overview of AI usage limitations
2024	University	Artificial Intelligence in Higher Education. Guidelines	Rīga Stradiņš University (RSU)	Latvia	Guide for academic staff and students on the use, limitation, and acquisition of AI in the study process.	GL	Consensus	B	ChatGPT, Midjourney, Turnitin, Plagiarism Check	Yes	Y	Y	Y	Ethical use of AI—Transparency—Continuous evaluation and feedback	Guidelines are comprehensive, covering ethical considerations, transparency and practical applications of AI.
2023	University	Recomendaciones para el uso de la IA generativa en la docencia	Universidad Autónoma de México UNAM	Mexico	Recommendations for the responsible and effective use of generative AI in education, ensuring ethical practices and enhancing learning outcomes	GL	Consensus	B	ChatGPT	Yes	Y	Y	Y	Ethical and responsible use of generative AI in education is crucial for maintaining academic integrity, generative AI tools should be used as supplementary aids, not as replacements for human effort and critical thinking, continuous dialogue and reflection on the use of AI in educational settings can enhance learning and innovation	Emphasises the importance of transparency and accountability in the use of AI tools, recommending that users document and explain their use of AI in their academic work. Highlights the need for ongoing education and support for both students and educators in effectively integrating AI into the learning process.
2024	University	Use of Generative‐Artificial Intelligences and Autonomous Content Generation in Learning and Teaching policy	University of Otago	New Zealand	Outline the university's approach to the use of generative‐artificial intelligences in learning and teaching	GL	ND	B	ChatGPT, DALL‐E	Assist learning and teaching	AM	N	Y	Ethical use, academic integrity, human agency in decision‐making	Emphasises responsible usage without banning
2023	University	Leveraging Generative Artificial Intelligence for Learning	University of Pretoria	South Africa	Guide students on the use of generative AI for learning	POL	ND	B	Various generative AI tools	Learning assistance	—	Y	N	Ethical use, maximising learning potential, responsible engagement with AI tools	Practical focus on enhancing learning through AI
2023	University	AI in Teaching and Learning at Wits	University of the Witwatersrand	South Africa	Promote academic integrity and innovative use of AI in teaching	GL	ND	B	ChatGPT	Various educational purposes	—	Y	Y	Ethical use, innovative teaching, academic integrity	Emphasises creativity and integrity in using AI
2024	University	Generative AI and teaching—Advice for educators	Karolinska Institute	Sweden	Advice on the use of generative AI in teaching	REC	ND	B	—	Assist in learning and teaching	MI	Y	Y	AI literacy, ethical and responsible use, academic integrity	Focus on practical advice and ethical considerations
2023	University	Leitlinien zum Umgang mit Künstlicher Intelligenz in der Lehre	University of Bern	Switzerland	Guide the use of AI in education	GL	ND	B	—	Allowed with specific conditions	MI	N	Y	Accept and critically engage with AI, define use cases and limitations, ensure integrity and originality in academic work	Reflects on dynamic and evolving nature of AI in education. Violations may be considered breaches of academic integrity
2024	University	Recommendations on the Use of Generative Artificial Intelligence at UZH	University of Zurich	Switzerland	Guide the use of generative AI in teaching and learning	GL	ND	B	ChatGPT	—	—	N	N	Faculties are responsible for creating specific guidelines, importance of transparency and fairness in assessments	Focuses on the importance of fairness and transparency
2024	University	King's guidance on generative AI for teaching, assessment and feedback	King's College London	UK	Guidance on the use of generative AI in teaching, assessment, and feedback	GL	Consensus	B	—	—	—	N	N	Clarification on AI use in teaching and assessment, emphasis on ethical use, guidelines for academic integrity	
2023	University	Staff Guide to Generative AI—Queen Mary Academy	Queen Mary University of London	UK	Guide on using GenAI in teaching	POL	ND	A	—	—	—	N	N	Explains how GenAI works, discusses strengths and limitations, considers ethical factors	Explores pedagogical, security and ethical factors
2023	University	Using AI tools in assessment	University College London	UK	Guide students on expectations and provide a three‐tier categorisation of AI use in assessments	REC	Consensus	B	—	Defined based on category	AM	Y	Y	Clarifies AI use in assessments, emphasises the need for clear communication, considers AI use as integral to certain assessments	Not specified
2024	University	Generative Artificial Intelligence and its Role Within Teaching, Learning and Assessment	University of Birmingham	UK	Guide the use of AI in teaching, learning, and assessment	GL	ND	B	ChatGPT, DALL‐E	Enhancing teaching and learning	UA	N	N	Ethical use of AI, enhance learning experiences, clear guidelines for usage	Focus on academic integrity
2024	University	Artificial Intelligence Policies, Guidelines and Considerations	Duke University	USA	Provide comprehensive policies and guidelines on AI use	GL	ND	B	—	Enhance educational experiences	UA	Y	N	Comprehensive policy coverage, emphasis on ethical use, integration into learning environments	Extensive coverage of AI considerations
2024	University	Generative Artificial Intelligence (AI) Guidelines	Harvard University	USA	Guide for the use of AI tools in academic contexts	GL	ND	B	ChatGPT, DALL‐E	Enhancing teaching and learning	UA	N	N	Ethical use of AI, support for academic activities, guidelines for responsible use	Emphasis on maintaining academic integrity
2024	University	Frequently asked questions about Teaching and AI	New York University	USA	Answer common questions about teaching with AI	POL	ND	B	—	Exploring generative AI, partnering with students, adapting assignments	CH	Y	Y	Opportunities of AI in teaching, communication of AI policies to students, adapting assignments to incorporate AI	Practical FAQ format
2024	University	Policy on use of Artificial Intelligence (AI) in assessments and deliverables	University of Californai San Fransico	USA	Provide guidelines on AI use in assessments and deliverables	GL	ND	B	None mentioned	Restricted use in assessments and deliverables	UA	Y	Y	Clear guidelines on AI usage in assessments, emphasis on documentation and supervision, delineation of prohibited uses	Focuses on academic integrity
2023	University	U‐M guidance for Faculty/Instructors	University of Michigan	USA	Guide faculty on integrating GenAI in teaching	GL	ND	A	U‐M GPT	Ethical use, enhancing teaching, and learning	S‐I	Y	N	Ethical and effective use, importance of transparency with students, adapt teaching strategies	Recommendations to adapt courses to AI integration
2023	University	U‐M guidance for students	University of Michigan	USA	Guide students on using GenAI tools	GL	ND	S	ChatGPT, DALL‐E 3, Gemini, Duet AI, Bing AI, Microsoft 365 Copilot, Bedrock, CodeWhisperer	Private and secure use for educational purposes	MI	Y	N	Use university‐provided tools for privacy, understand limitations and biases, ethical use of AI tools	Practical tips for safe and ethical AI use
2023	University	Teaching Generative AI Guidance	University of North Carolina	USA	Guide integrating GenAI in teaching and learning	GL	ND	A	—	Use for enhancing learning experiences	MI	Y	Y	Ethical use of AI, support for academic activities, guidelines for responsible use	Emphasises the integration of AI in education
2024	University	Artificial Intelligence Interim Guidelines	University of Washington	USA	Provide interim guidelines for AI use in education	REC	ND	B	—	Educational enhancement	MI	Y	Y	Interim nature and need for updates, balancing AI benefits and risks, ensuring academic integrity	Focus on continuous updates and feedback

Abbreviations: AM, academic misconduct; CH, cheating or inappropriate use; MI, misuse (e.g., leading to bias, privacy issues, dishonesty or violations of academic integrity); S‐I, sharing sensitive information; UA, unauthorised use (e.g., in assessments, scholarly work or deliverables); VC, verbatim copying prohibited; Y, yes (explicitly prohibited).

^a^ Type: GL for guideline; REC for recommendation; POL for policy.

^b^ Method: ND for no declared.

^c^ Target audience: A for academic staff, B for both and S for students.

^d^ Prohibited uses of GenAI: ‐: not mentioned or none specified.

## 4 Discussion

This scoping review found several guidelines from universities with dental schools that guide using GenAI for academic staff. The thematic analysis revealed common themes and specific focus areas for integrating GenAI into education. Previously, a global survey of dental educators revealed varying perceptions of AI's impact on dental education, with some countries like Italy and Norway anticipating little or no impact, while others like Estonia, South Africa, Japan and Turkey expecting significant changes [3]. The main barriers to AI implementation in dental education included resistance to change, knowledge gaps, fears of misuse, cost, accessibility, validity, reliability, mindset, ethics and challenges such as risks of plagiarism, bias or lack of transparency [3].

This review's key themes included ethical use, transparency and academic integrity. Universities like the University of Michigan, Harvard and Zurich emphasised responsible AI use to enhance teaching while maintaining integrity. These observations are based on thematic analysis and may involve subjective interpretation of available content. The University of Sydney and PUC highlighted transparency. Among organisations, the European University Association, SARUA and UNESCO focused on responsible AI use, emphasising ethical engagement and addressing challenges. UNESCO promoted informed AI use in education. Notably, dental education bodies like ADEA and ADEE lack comprehensive AI guidelines, mentioning only professional use without specific recommendations for dental education. This gap highlights the need for detailed guidance to effectively integrate GenAI into dental education. Despite the lack of comprehensive AI guidelines from dental education bodies, dental educators can still take proactive steps to integrate GenAI into their curriculum. The first step for dental educators looking to integrate GenAI into their curriculum is to check if their institution has existing policies on AI usage. If no such policies exist, starting with the ‘UNESCO ChatGPT and AI in Higher Education Quick Start Guide’ is advisable, followed by the ‘UNESCO Guidance for GenAI in Education and Research’. For developing comprehensive guidelines or policies, the UNESCO document by Miao et al. [10] offers valuable recommendations for policymakers. These resources provide foundational guidance on AI's ethical and effective use in educational settings, ensuring alignment with best practices and regulatory standards.

The potential impact of AI in education shows that technology‐enhanced teaching and assessment methods can improve learning outcomes, particularly in health sciences and dentistry [1, 11]. Generative AI enables personalised educational experiences by adapting content to individual student needs and providing targeted feedback. These tools can simulate complex diagnostic scenarios [12], allowing students to refine clinical decision‐making in safe, virtual environments. However, their use necessitates careful oversight to mitigate risks such as misinformation [13] and automation bias [14]. The ethical use of AI remains a significant concern, particularly regarding data privacy and academic integrity. Generative AI introduces unique challenges, such as the potential for ‘hallucinated’ information [15], requiring robust guidelines to ensure accurate and trustworthy outputs in educational contexts. Recent recommendations emphasise fostering AI literacy among students and faculty as a key strategy to address these issues effectively [10]. Most analysed documents agreed on these advantages and disadvantages.

This scoping review has limitations. As an exploratory study, some relevant information may have been missed. Many institutions have policies and documents on intranets, which may not be publicly accessible. Additionally, finding relevant information on some academic websites was challenging due to the nature of hyperlinks, and some institutions with public policies may have been missed due to a lack of indexing. Furthermore, despite sending mailings to over 400 educators, the response rate was low, limiting the breadth of the data collected. Some documents were provided in PDF format, facilitating reference for faculty and students. However, other documents were dispersed across multiple web pages, complicating access. To improve guidance usability and discoverability, institutions should provide consolidated information either on a single web page or as a downloadable PDF. The PUC and Riga Stradins University (RSU) offer good examples of effective presentations. Additionally, while providing valuable insights, the thematic analysis is inherently subjective and reflects the perspectives of the reviewers analysing the guideline contents. Although steps were taken to minimise bias, such as independent reviewer input, this subjectivity remains a limitation of this study.

The content of the guidelines varied significantly. Based on common issues, a comprehensive guideline should include clear policies on ethical and responsible AI usage, transparency in AI application, academic integrity, detailed supervision and documentation procedures. Guidelines should also specify permitted and prohibited uses of AI tools and include actionable penalties for misuse.

Implementing GenAI in dental education involves significant costs. Harvard and the University of Michigan have created secure environments or sandboxes for faculty to use LLMs while respecting copyright and data security policies. Similar to the integration of IT during the COVID‐19 pandemic, a proactive and coordinated approach is essential to address barriers such as infrastructure limitations, cost disparities and resistance to change. Universities like Harvard and Michigan exemplify best practices by providing secure AI sandboxes for academic use. Another cost to consider is the implementation of tools for the academic staff to detect GenAI misuse. The guidelines revealed that GenAI's most commonly prohibited uses included unauthorised use as original work, misuse leading to academic dishonesty, inappropriate use in assessments and sharing sensitive personal information. Institutions like the University of Michigan, the University of Pretoria and the University of Otago emphasised the prohibition of using AI‐generated content without proper citation and the potential for misuse in academic work. Universities proposed using AI detectors like GPTZero or plagiarism detectors like Turnitin. However, dealing with false positives and negatives remains challenging [16]. The best way to prevent AI misuse is with AI literacy [17]. To ensure a comprehensive understanding and responsible use of AI, it is essential to incorporate courses covering AI fundamentals, ethical implications and practical applications in dentistry [18]. Additionally, the integration of GenAI will impact the formal assessment of quizzes, tests, essays and theses [3], necessitating reevaluating the assessment methods. Practical and clinical areas of the dental curriculum may remain relatively unaffected, but implementing GenAI presents an opportunity to shift the focus from data delivery to cultivating students' critical thinking skills, enabling them to identify limitations and inaccuracies in AI‐generated outputs.

Universities should implement GenAI with the urgency demonstrated during the COVID‐19 pandemic when they rapidly adopted technological advancements in education. This approach will help institutions stay ahead in integrating cutting‐edge technologies into their teaching practices. For instance, IT implementation was recommended in previous years [19], but many universities cited needing more resources, infrastructure, or training as barriers [20]. Before COVID‐19, students primarily used social media to find dental content and resources [21]. Still, it was only during the pandemic that dental educators gained experience and confidence in using IT, accelerating the adoption and innovation of e‐learning [22]. By 2020, 90% of European dental schools used online pedagogical tools, with 72% utilising live or streamed video and online materials [23]. The advent of GenAI should also be an urgent call for re‐evaluating the dental curriculum. This re‐evaluation will ensure alignment between the dental profession, patient needs and public institutions [24]. Recent reports highlight a mismatch between the number of dentists and the population's oral health [25], underscoring the need for a comprehensive and modern approach to dental education [26]. For instance, reports on early childhood caries prevalence have shown little change in the last 20 years [27], despite numerous technological innovations.

After reviewing the available documents, three key areas emerged for integrating GenAI technologies into dental education. First, pedagogical changes are needed, with dental schools rethinking assessments to prioritise understanding, critical thinking and problem‐solving over AI‐generated information. Curricula should integrate AI education to promote efficiency, critical thinking and ethics [18] while establishing policies to prevent misuse and maintain academic integrity. Second, in terms of governance, most policies and guidelines should be implemented at the university level to ensure consistency across disciplines. These can address ethical concerns, privacy and security, maintaining transparency and accountability. While dental‐specific policies are valuable, broader university‐wide or national AI guidelines ensure consistency across disciplines. Such an approach allows for cohesive governance, enabling dental schools to tailor applications to their unique requirements. Continuous monitoring and evaluation of AI technologies are necessary to ensure effectiveness and address arising issues. Finally, on an operational level, AI literacy training and support for faculty, staff and students are essential. Resource investment is needed to equip all stakeholders with the knowledge and skills to use AI technologies responsibly and effectively. For example, universities like Harvard and Michigan offer secure environments or sandboxes, allowing faculty to use LLMs in classrooms while adhering to copyright and data security policies.

Based on the common issues identified in various guidelines, policies and recommendations, dental institutions should address the common issues identified in various guidelines, policies and recommendations. Key elements include establishing clear AI policies, offering AI courses, investing in AI infrastructure, forming a multidisciplinary committee, conducting regular policy reviews and fostering open dialogue. Table 2 presents more details on these elements.

**TABLE 2:** Formulating Gen AI guidelines for dental institutions: Considerations and actionable recommendations derived from current guidance.

Item	Actionable point	Implementation level	Detail
Clear AI Policies	Develop and implement clear policies and guidelines for using generative AI in education.	University	These policies should ensure ethical and responsible usage while being flexible enough to encourage creativity. Policies can be customised at the course level, allowing faculty to define AI usage according to specific learning objectives. These policies should be easily accessible, preferably in PDF and scrollable HTML, with all relevant information on one page.
AI Literacy	Offer AI literacy courses.	University	Preferable at the university level. This would benefit a wider range of students and ensure that foundational AI knowledge is taught consistently across disciplines.
	Incorporate AI modules within dental courses.	Faculty or School	For more specialised applications, dental‐specific AI modules can be integrated into the dental curriculum, focusing on the practical applications of AI in dentistry, such as diagnostic support, clinical decision‐making and ethics in dental AI usage.
	Continue staff education on AI.	University	Ongoing professional development for faculty and staff should include AI literacy to ensure they can critically evaluate AI technologies, stay informed about new AI developments and integrate AI effectively into their teaching practices.
AI Infrastructure Investment	Invest in infrastructure and resources to support integrating AI technologies in dental education.	University	This investment will ensure that students and staff can access the tools and platforms to utilise AI effectively and safely in their educational practices.
AI Multidisciplinary Committee	Create a multidisciplinary advisory group to guide AI integration.	University	The group should serve as an advisory body, offering guidance on AI integration to support educational goals, encourage innovation and address ethical concerns without restricting creativity instead of overseeing and ‘policing’ AI usage.
Regular AI Policy Review	Regularly review and update AI policies to keep pace with technological advancements and regulatory changes.	University	This review will ensure the institution's AI policies remain relevant and effective.

## 5 Conclusion

This scoping review identified guidelines from universities and organisations emphasising ethical use, transparency and academic integrity for GenAI in education. These findings are based on thematic analysis, highlighting a need for further exploration and validation by dental education bodies, such as ADEE and ADEA. GenAI offers potential benefits like personalised learning and efficient content creation, but challenges such as biases and ethical concerns must be managed. Guidelines should include clear policies on AI usage, specify permitted and prohibited uses and detail penalties for misuse. Implementing GenAI in dental education requires substantial investments in secure environments and AI misuse detection tools. It also necessitates reevaluating traditional assessment methods and emphasising critical thinking skills and ethical AI use. To stay innovative, universities should implement GenAI with the same urgency as other technological innovations, as with the COVID‐19 response. By addressing common issues and providing actionable recommendations, educational associations like ADEE and ADEA can play a crucial role in effectively deploying and implementing AI technologies into curricula aligning dental education with professional requirements and societal needs.

## Author Contributions

Conceptualization: S.E.U., F.S. and I.M. Data curation, formal analysis and project administration: S.E.U. Funding acquisition: S.E.U. and I.M. Investigation: S.E.U., I.M. and F.S. Methodology: S.E.U. and I.M. Writing – original draft: S.E.U. Writing – review and editing: S.E.U., I.M. and F.S.

## Conflicts of Interest

The authors declare no conflicts of interest.

## Supporting information


Table S1.

Table S2.

## Data Availability

The manuscript's tables and Tables S1 and S2 contain all data supporting this review's results.
