# Supplementary material for: Integrating Generative AI in Dental Education: A Scoping Review of Current Practices and Recommendations
Source: Eur J Dent Educ. 2025 Jan 31;29(2):341–55. doi: 10.1111/eje.13074 (PMC12006694; doi:10.1111/eje.13074)
Supplement: Supplementary file 1 — Table S1. [file EJE-29-341-s001.docx]

**Supplementary Table 1**. Sites screened and included.

| **N** | **Reason for screening** | **Type (*)** | **University** | **City** | **Country** | **Continent** | **URL for search** | **Status** | **Language** | **Document found** | **URL Site** | **Search strategy** |
| --- | --- | --- | --- | --- | --- | --- | --- | --- | --- | --- | --- | --- |
| 1 | QS Ranking | U | The University of Melbourne | Parkville | Australia | Oceania | <https://www.unimelb.edu.au/> | Available | English | Yes | https://academicintegrity.unimelb.edu.au/plagiarism-and-collusion/artificial-intelligence-tools-and-technologies / https://academicintegrity.unimelb.edu.au/plagiarism-and-collusion/artificial-intelligence-tools-and-technologies | site:https://www.unimelb.edu.au/ (generative AI OR "generative artificial intelligence" OR chatGPT) (guidance OR guideline OR Recommendations OR implementation OR policy) |
| 2 | QS Ranking | U | The University of Hong Kong | Hong Kong | Hong Kong SAR | Asia | <https://www.hku.hk/> | Available | English | Yes | <https://aied.talic.hku.hk/aipolicy/> | site:https://www.hku.hk/ (generative AI OR "generative artificial intelligence" OR chatGPT) (guidance OR guideline OR Recommendations OR implementation OR policy) |
| 3 | QS Ranking | U | University of Michigan-Ann Arbor | Ann Arbor | United States | North America | <https://umich.edu/> | Available | English | Yes | <https://genai.umich.edu/guidance> | site:https://umich.edu/ (generative AI OR "generative artificial intelligence" OR chatGPT) (guidance OR guideline OR Recommendations OR implementation OR policy) |
| 4 | QS Ranking | U | Harvard University | Cambridge | United States | North America | <https://www.harvard.edu/> | Available | English | Yes | https://harvard.edu/ai/ and https://huit.harvard.edu/ai/guidelines | site:https://www.harvard.edu/ (generative AI OR "generative artificial intelligence" OR chatGPT) (guidance OR guideline OR Recommendations OR implementation OR policy) |
| 5 | QS Ranking | U | University of Washington | Seattle | United States | North America | <https://www.washington.edu/> | Available | English | Yes | <https://itconnect.uw.edu/guides-by-topic/security-authentication/artificial-intelligence-guidelines/?_gl=1*1uhvk76*_ga*OTYxMzM2NDE1LjE3MTkyNDM5NjM.*_ga_3T65WK0BM8*MTcxOTMzOTQxMy4xLjEuMTcxOTMzOTQzNi4wLjAuMA..*_ga_JLHM9WH4JV*MTcxOTMzOTQxMy4xLjEuMTcxOTMzOTQzNi4wLjAuMA..> | site:https://www.washington.edu/ (generative AI OR "generative artificial intelligence" OR chatGPT) (guidance OR guideline OR Recommendations OR implementation OR policy) |
| 6 | QS Ranking | U | University of California, San Francisco | San Francisco | United States | North America | <https://www.ucsf.edu/> | Available | English | Yes | <https://pharm.ucsf.edu/current/policies/ai> | site:https://www.ucsf.edu/ (generative AI OR "generative artificial intelligence" OR chatGPT) (guidance OR guideline OR Recommendations OR implementation OR policy) |
| 7 | QS Ranking | U | University of North Carolina at Chapel Hill | Chapel Hill | United States | North America | <https://www.unc.edu/> | Available | English | Yes | <https://provost.unc.edu/teaching-generative-ai-guidance/> | site:https://www.unc.edu/ (generative AI OR "generative artificial intelligence" OR chatGPT) (guidance OR guideline OR Recommendations OR implementation OR policy) |
| 8 | QS Ranking | U | University of California, Los Angeles (UCLA) | Los Angeles | United States | North America | <https://www.ucla.edu/> | Available | English | Yes | <https://teaching.ucla.edu/resources/ai_guidance/> | site:https://www.ucla.edu/ (generative AI OR "generative artificial intelligence" OR chatGPT) (guidance OR guideline OR Recommendations OR implementation OR policy) |
| 9 | QS Ranking | U | University of Birmingham | Birmingham | United Kingdom | Europe | <https://www.birmingham.ac.uk/> | Available | English | Yes | <https://www.birmingham.ac.uk/university/hefi/gai> | site:https://www.birmingham.ac.uk/ (generative AI OR "generative artificial intelligence" OR chatGPT) (guidance OR guideline OR Recommendations OR implementation OR policy) |
| 10 | QS Ranking | U | King's College London | London | United Kingdom | Europe | <https://www.kcl.ac.uk/> | Available | English | Yes | <https://www.kcl.ac.uk/about/strategy/learning-and-teaching/ai-guidance/macro-level> | site:https://www.kcl.ac.uk/ (generative AI OR "generative artificial intelligence" OR chatGPT) (guidance OR guideline OR Recommendations OR implementation OR policy) |
| 11 | QS Ranking | U | KU Leuven | Leuven | Belgium | Europe | <https://www.kuleuven.be/english> | Available | English | Yes | <https://www.kuleuven.be/english/education/leuvenlearninglab/support/highlighted/generative-artificial-intelligence> | site:https://www.kuleuven.be/english (generative AI OR "generative artificial intelligence" OR chatGPT) (guidance OR guideline OR Recommendations OR implementation OR policy) |
| 12 | QS Ranking | U | New York University (NYU) | New York City | United States | North America | <https://www.nyu.edu/> | Available | English | Yes | <https://www.nyu.edu/faculty/teaching-and-learning-resources/teaching-with-generative-tools/frequently-asked-questions.html#5> | site:https://www.nyu.edu/ (generative AI OR "generative artificial intelligence" OR chatGPT) (guidance OR guideline OR Recommendations OR implementation OR policy) |
| 13 | Colleague | U | University of Oulu | Oulu | Finland | Europe | [https://www.oulu.fi/](https://www.oulu.fi/en) | Available | English | Yes | <https://www.oulu.fi/en/for-students/studying-university/guidelines-use-artificial-intelligence-education#:~:text=Guidelines%20of%20the%20University%20of,use%20AI%20%E2%80%93%20check%20if%20necessary!> | https://www.oulu.fi/ (generative AI OR "generative artificial intelligence" OR chatGPT) (guidance OR guideline OR Recommendations OR implementation OR policy) |
| 14 | QS Ranking | U | Queen Mary University of London | London | United Kingdom | Europe | <https://www.qmul.ac.uk/> | Available | English | Yes | <https://www.qmul.ac.uk/queenmaryacademy/educators/resources/assessment-and-feedback/resources/generative-ai-and-chat-gpt/> | site:https://www.qmul.ac.uk/ (generative AI OR "generative artificial intelligence" OR chatGPT) (guidance OR guideline OR Recommendations OR implementation OR policy) |
| 15 | Colleague | U | Riga Stradins University | Riga | Latvia | Europe | <https://www.rsu.lv/en> | Available | English | Yes | <https://www.rsu.lv/maksligais-intelekts-augstakaja-izglitiba> | site:https://www.rsu.lv/en (generative AI OR "generative artificial intelligence" OR chatGPT) (guidance OR guideline OR Recommendations OR implementation OR policy) |
| 16 | QS Ranking | U | Tokyo Medical and Dental University (TMDU) | Tokyo | Japan | Asia | <https://www.tmd.ac.jp/english/> | Available | English | Yes | <https://www.tmd.ac.jp/cmn/rules/houki/7hen/4shou/2setsu/74208aiguideline.pdf> | site:https://www.tmd.ac.jp/english/ (generative AI OR ¨generative artificial intelligence" OR chatGPT) (guidance OR guideline OR Recommendations OR implementation OR policy) |
| 17 | QS Ranking | U | UCL | London | United Kingdom | Europe | <https://www.ucl.ac.uk/> | Available | English | Yes | <https://www.ucl.ac.uk/teaching-learning/generative-ai-hub/using-ai-tools-assessment> | site:https://www.ucl.ac.uk/ (generative AI OR "generative artificial intelligence" OR chatGPT) (guidance OR guideline OR Recommendations OR implementation OR policy) |
| 18 | QS Ranking | U | Karolinska Institutet | Stockholm | Sweden | Europe | <https://www.ki.se/en> | Available | English | Yes | <https://www.unibe.ch/unibe/portal/content/e809/e878/e880/e915/e36136/e1368980/e1383230/20230516_LeitlinienKI_datiert_ger.pdf> | site:https://www.ki.se/en (generative AI OR "generative artificial intelligence" OR chatGPT) (guidance OR guideline OR Recommendations OR implementation OR policy) |
| 19 | QS Ranking | U | University of Bern | Bern | Switzerland | Europe | <https://www.unibe.ch/university/index_eng.html> | Available | English | Yes | <https://www.unibe.ch/unibe/portal/content/e977779/e981607/e981611/pane981622/e981624/HDBro_2024_V5_ger.pdf> | site:https://www.unibe.ch/university/index_eng.html (generative AI OR "generative artificial intelligence" OR chatGPT) (guidance OR guideline OR Recommendations OR implementation OR policy) |
| 20 | QS Ranking | U | University of Zurich | Zürich | Switzerland | Europe | <https://www.uzh.ch/en.html> | Available | English | Yes | <https://www.uzh.ch/en/explore/basics/ai/recommendations.html> | site:https://www.uzh.ch/en.html (generative AI OR "generative artificial intelligence" OR chatGPT) (guidance OR guideline OR Recommendations OR implementation OR policy) |
| 21 | QS Ranking | U | Academic Centre for Dentistry Amsterdam (ACTA) | Amsterdam | Netherlands | Europe | <https://www.acta.nl/en> | Available | English | No | No | site:https://www.acta.nl/en (generative AI OR "generative artificial intelligence" OR chatGPT) (guidance OR guideline OR Recommendations OR implementation OR policy) |
| 22 | QS Ranking | U | University of Gothenburg | Gothenburg | Sweden | Europe | [https://www.gu.se/](https://www.gu.se/english) | Available | English | No | No | site:https://www.gu.se/ (generative AI OR "generative artificial intelligence" OR chatGPT) (guidance OR guideline OR Recommendations OR implementation OR policy) |
| 23 | QS Ranking | U | Sichuan University | Chengdu | China (Mainland) | Asia | <https://www.scu.edu.cn/en/> | Available | English | No | No | site:https://www.scu.edu.cn/en/ (generative AI OR "generative artificial intelligence" OR chatGPT) (guidance OR guideline OR Recommendations OR implementation OR policy) |
| 24 | QS Ranking | U | Universidade de São Paulo | São Paulo | Brazil | South America | <https://www.usp.br/en/> | Available | English | No | No | site:https://www.usp.br/en/ (generative AI OR "generative artificial intelligence" OR chatGPT) (guidance OR guideline OR Recommendations OR implementation OR policy) |
| 25 | QS Ranking | U | Imam Abdulrahman Bin Faisal University (IAU) | Dammam | Saudi Arabia | Asia | <https://www.iau.edu.sa/en> | Available | English | No | No | site:https://www.iau.edu.sa/en (generative AI OR "generative artificial intelligence" OR chatGPT) (guidance OR guideline OR Recommendations OR implementation OR policy) |
| 26 | QS Ranking | U | Complutense University of Madrid | Madrid | Spain | Europe | <https://www.ucm.es/english> | Available | English | No | No | site:https://www.ucm.es/english (generative AI OR "generative artificial intelligence" OR chatGPT) (guidance OR guideline OR Recommendations OR implementation OR policy) |
| 27 | QS Ranking | U | University of Pennsylvania | Philadelphia | United States | North America | <https://www.upenn.edu/> | Available | English | No | No | site:https://www.upenn.edu/ (generative AI OR "generative artificial intelligence" OR chatGPT) (guidance OR guideline OR Recommendations OR implementation OR policy) |
| 28 | QS Ranking | U | Universidade Estadual de Campinas (Unicamp) | Campinas | Brazil | South America | <https://www.unicamp.br/unicamp/en> | Available | English | No | No | site:https://www.unicamp.br/unicamp/en (generative AI OR "generative artificial intelligence" OR chatGPT) (guidance OR guideline OR Recommendations OR implementation OR policy) |
| 29 | QS Ranking | U | Saveetha Institute of Medical And Technical Sciences (SIMATS) | Tiruvallur | India | Asia | <https://www.saveetha.com/> | Available | English | No | No | site:https://www.saveetha.com/ (generative AI OR "generative artificial intelligence" OR chatGPT) (guidance OR guideline OR Recommendations OR implementation OR policy) |
| 30 | QS Ranking | U | Tufts University | Medford | United States | North America | <https://www.tufts.edu/> | Available | English | No | No | site:https://www.tufts.edu/ (generative AI OR "generative artificial intelligence" OR chatGPT) (guidance OR guideline OR Recommendations OR implementation OR policy) |
| 31 | QS Ranking | U | Universidad de Chile | Santiago | Chile | South America | <https://www.uchile.cl/en/> | Available | English | No | No | site:https://www.uchile.cl/en/ (generative AI OR "generative artificial intelligence" OR chatGPT) (guidance OR guideline OR Recommendations OR implementation OR policy) |
| 32 | QS Ranking | U | National University of Singapore (NUS) | Singapore | Singapore | Asia | <https://www.nus.edu.sg/> | Available | English | No | No | site:https://www.nus.edu.sg/ (generative AI OR "generative artificial intelligence" OR chatGPT) (guidance OR guideline OR Recommendations OR implementation OR policy) |
| 33 | Colleague | U | LMU | Munich | Germany | Europe | <https://www.en.uni-muenchen.de/> | Available | English | No | No | site:https://www.en.uni-muenchen.de/ (generative AI OR "generative artificial intelligence" OR chatGPT) (guidance OR guideline OR Recommendations OR implementation OR policy) |
| 34 | Colleague | U | Cardiff University | Cardiff | Wales, UK | Europe | <https://www.cardiff.ac.uk/> | Available | English | No | No | site:https://www.cardiff.ac.uk/ (generative AI OR "generative artificial intelligence" OR chatGPT) (guidance OR guideline OR Recommendations OR implementation OR policy) |
| 35 | Google Search | U | University of Sidney | Sidney | Australia | Oceania | <https://www.sydney.edu.au/> | Available | English | Yes | <https://educational-innovation.sydney.edu.au/teaching@sydney/frequently-asked-questions-about-generative-ai-at-sydney/> | site:https://www.sydney.edu.au/ (generative AI OR "generative artificial intelligence" OR chatGPT) (guidance OR guideline OR Recommendations OR implementation OR policy) |
| 36 | Google Search | U | Duke University | Durham | United States | North America | <https://duke.edu/> | Available | English | Yes | <https://learninginnovation.duke.edu/ai-and-teaching-at-duke-2/artificial-intelligence-policies-in-syllabi-guidelines-and-considerations/> | site:https://duke.edu/ (generative AI OR "generative artificial intelligence" OR chatGPT) (guidance OR guideline OR Recommendations OR implementation OR policy) |
| 37 | Google Search | U | University of the Witwatersrand | Johannesburg | South Africa | Africa | <https://www.wits.ac.za/> | Available | English | Yes | <https://www.wits.ac.za/media/wits-university/learning-and-teaching/cltd/documents/AI-in-teaching-and-learning-at-Wits.pdf> | site:https://www.wits.ac.za/ (generative AI OR "generative artificial intelligence" OR chatGPT) (guidance OR guideline OR Recommendations OR implementation OR policy) |
| 38 | Colleague | U | University of Pretoria |  | South Africa | Africa | <https://www.up.ac.za> | Available | English | Yes | <https://www.up.ac.za/media/shared/391/pdfs/up-student-guide_-leveraging-generative-artificial-intelligence-for-learning.zp242396.pdf> | site:https://www.up.ac.za (generative AI OR "generative artificial intelligence" OR chatGPT) (guidance OR guideline OR Recommendations OR implementation OR policy) |
| 39 | Google Search | U | Pontificia Universidad Catolica | Santiago | Chile | South America | <https://www.uc.cl/> | Available | English | Yes | <https://docencia.ia.uc.cl/> | site:https://www.uc.cl/ (generative AI OR "generative artificial intelligence" OR chatGPT) (guidance OR guideline OR Recommendations OR implementation OR policy) |
| 40 | Google Search | U | Universidad Autonoma de Mexico | Ciudad de Mexico | Mexico | North America | <https://www.unam.mx/> | Available | Spanish | Yes | <https://cuaed.unam.mx/descargas/recomendaciones-uso-iagen-docencia-unam-2023.pdf> | site:https://www.unam.mx/ (generative AI OR "generative artificial intelligence" OR chatGPT) (guidance OR guideline OR Recommendations OR implementation OR policy) |
| 1 | Google Search | AO | ADEE: Association for Dental Education in Europe |  |  | Europe | <https://adee.org/> | Available | English | No | No | site:https://adee.org/ (generative AI OR "generative artificial intelligence" OR chatGPT) (guidance OR guideline OR Recommendations OR implementation OR policy) |
| 2 | Google Search | AO | ADEA: American Dental Education Association |  |  | North America | <https://www.adea.org/> | Available | English | No | No | site:https://www.adea.org/ (generative AI OR "generative artificial intelligence" OR chatGPT) (guidance OR guideline OR Recommendations OR implementation OR policy) |
| 3 | Google Search | AO | ADSA: American Student Dental Association |  |  | North America | <https://www.asdanet.org/> | Available | English | No | No | site:https://www.asdanet.org/ (generative AI OR "generative artificial intelligence" OR chatGPT) (guidance OR guideline OR Recommendations OR implementation OR policy) |
| 4 | Google Search | AO | EUA: European University Association’s Learning and Teaching Steering Committee |  |  | Europe | [https://eua.eu/](https://eua.eu/resources/publications/1059:artificial-intelligence-tools-and-their-responsible-use-in-higher-education-learning-and-teaching.html) | Available | English | Yes | <https://eua.eu/downloads/publications/position_ai%20in%20lt.pdf> | site:https://eua.eu/ (generative AI OR "generative artificial intelligence" OR chatGPT) (guidance OR guideline OR Recommendations OR implementation OR policy) |
| 5 | Google Search | AO | SARUA: Southern African Regional Universities Association |  |  | Africa | <https://sarua.africa/> | Available | English | Yes | <https://sarua.africa/guidance-on-the-use-of-chatgpt-by-lecturers-and-students/> | site:https://sarua.africa/ (generative AI OR "generative artificial intelligence" OR chatGPT) (guidance OR guideline OR Recommendations OR implementation OR policy) |
| 1 | Google Search | AO | UNESCO |  |  | Global | <https://www.unesco.org/en> | Available | English | Yes | <https://www.unesco.org/en/articles/guidance-generative-ai-education-and-research> | site:https://www.unesco.org/en (generative AI OR "generative artificial intelligence" OR chatGPT) (guidance OR guideline OR Recommendations OR implementation OR policy) |
| * Type | | | | | | | | | | | | |
| U= University | | | | | | | | | | | | |
| AO = Association or Organization | | | | | | | | | | | |  |
