# Supplementary material for: Integrating Generative AI in Dental Education: A Scoping Review of Current Practices and Recommendations
Source: Eur J Dent Educ. 2025 Jan 31;29(2):341–55. doi: 10.1111/eje.13074 (PMC12006694; doi:10.1111/eje.13074)
Supplement: Supplementary file 2 — Table S2. [file EJE-29-341-s002.docx]

**Supplementary Table 2**. Sites screened and excluded.

| **ID** | **Exclusion reason** | **Document** | **Year** | **Name** | **Authors** | **Institution** | **Country** |
| --- | --- | --- | --- | --- | --- | --- | --- |
| 2 | Duplicated | N/A (PDF) | 2023 | Leitlinien zum Umgang mit Künstlicher Intelligenz in der Lehre | Vizerektorat Lehre, Generalsekretariat | University of Bern | Switzerland |
| 11 | Duplicated | [Kings ai-guidance.html](https://www.kcl.ac.uk/about/strategy/learning-and-teaching/ai-guidance) | 2024 | King’s guidance on generative AI for teaching, assessment and feedback | King's College London | King's College London | UK |
| 15 | Duplicated | [Kings ai-guidance.html](https://www.kcl.ac.uk/about/strategy/learning-and-teaching/ai-guidance) | 2024 | King’s guidance on generative AI for teaching, assessment and feedback | King's College London | King's College London | UK |
| 13 | Duplicated | [Kings Macro-level_ University-wide principles and policy _ King's College London.html](https://www.kcl.ac.uk/about/strategy/learning-and-teaching/ai-guidance/macro-level) | 2024 | Macro-level: University-wide principles and policy | King's College London | King's College London | UK |
| 25 | Duplicated | [KU Leuven Learning Lab](https://www.kuleuven.be/english/education/leuvenlearninglab/support/highlighted/generative-artificial-intelligence) | 2023 | Responsible use of Generative Artificial Intelligence - KU Leuven Learning Lab | KU Leuven | KU Leuven | Belgium |
| 31 | Duplicated | [Responsible use of Generative Artificial Intelligence - Student](https://www.kuleuven.be/english/education/student/educational-tools/generative-artificial-intelligence) | 2024 | Responsible use of Generative Artificial Intelligence - Student | KU Leuven | KU Leuven | Belgium |
| 27 | Duplicated | [Queen Mary Academy Staff Guide](https://www.qmul.ac.uk/queenmaryacademy/educators/resources/assessment-and-feedback/resources/generative-ai-and-chat-gpt/) | 2023 | Staff Guide to Generative AI - Queen Mary Academy | Queen Mary Academy | Queen Mary University of London | UK |
| 28 | Duplicated | [UZH Guidelines](https://www.uzh.ch/en/explore/basics/ai/recommendations.html) | 2024 | Recommendations on the Use of Generative Artificial Intelligence at UZH | University of Zurich | University of Zurich | Switzerland |
| 24 | Duplicated | [UZH Recommendations](https://www.uzh.ch/en/explore/basics/ai/recommendations.html) | 2024 | Recommendations on the Use of Generative Artificial Intelligence at UZH | University of Zurich | University of Zurich | Switzerland |
| 18 | Duplicated | [Macro-level: University-wide principles and policy](https://www.kcl.ac.uk/about/strategy/learning-and-teaching/ai-guidance/macro-level) | 2024 | Macro-level: University-wide principles and policy | King's College London | King's College London | UK |
| 12 | Duplicated | [kings doctoral-assessment.html](https://www.kcl.ac.uk/about/strategy/learning-and-teaching/ai-guidance/doctoral-assessment) | 2024 | Generative AI: Guidance for doctoral students, supervisors, and examiners | King's College London | King's College London | UK |
| 17 | Duplicated | [Generative AI: Guidance for doctoral students, supervisors, and examiners](https://www.kcl.ac.uk/about/strategy/learning-and-teaching/ai-guidance/doctoral-assessment) | 2024 | Generative AI: Guidance for doctoral students, supervisors, and examiners | King's College London | King's College London |  |
| 19 | University | [Generative AI - Student guidance](https://www.kcl.ac.uk/about/strategy/learning-and-teaching/ai-guidance/student-guidance) | 2024 | Generative AI - Student guidance | King's College London | King's College London |  |
| 14 | University | [kings student-guidance.html](https://www.kcl.ac.uk/about/strategy/learning-and-teaching/ai-guidance/student-guidance) | 2024 | Generative AI - Student guidance | King's College London | King's College London |  |
| 14 | only for students | [kings student-guidance.html](https://www.kcl.ac.uk/about/strategy/learning-and-teaching/ai-guidance/student-guidance) | 2024 | Generative AI - Student guidance | King's College London | King's College London | UK |
| 19 | only for students | [Generative AI - Student guidance](https://www.kcl.ac.uk/about/strategy/learning-and-teaching/ai-guidance/student-guidance) | 2024 | Generative AI - Student guidance | King's College London | King's College London | UK |
| 8 | only for students | [U-M Guidance for Students](https://genai.umich.edu/guidance/students) | 2023 | U-M Guidance for Students | University of Michigan | University of Michigan | USA |
| 22 | only for students | [Policy on Use of Artificial Intelligence (AI) in Assessments and Deliverables](https://pharm.ucsf.edu/current/policies/ai) | 2024 | Policy on Use of Artificial Intelligence (AI) in Assessments and Deliverables | UCSF School of Pharmacy | UCSF School of Pharmacy | USA |
| 26 | only for students | [KU Leuven Student Guide](https://www.kuleuven.be/english/education/student/educational-tools/generative-artificial-intelligence) | 2024 | Responsible use of Generative Artificial Intelligence - Student | KU Leuven | KU Leuven | Belgium |
| 34 | only for students | [Policy on Use of Artificial Intelligence (AI) in Assessments and Deliverables](https://pharm.ucsf.edu/current/policies/ai) | 2024 | Policy on Use of Artificial Intelligence (AI) in Assessments and Deliverables | UCSF School of Pharmacy | UCSF School of Pharmacy | USA |
| 12 | only for students | [kings doctoral-assessment.html](https://www.kcl.ac.uk/about/strategy/learning-and-teaching/ai-guidance/doctoral-assessment) | 2024 | Generative AI: Guidance for doctoral students, supervisors, and examiners | King's College London | King's College London | UK |
| 17 | only for students | [Generative AI: Guidance for doctoral students, supervisors, and examiners](https://www.kcl.ac.uk/about/strategy/learning-and-teaching/ai-guidance/doctoral-assessment) | 2024 | Generative AI: Guidance for doctoral students, supervisors, and examiners | King's College London | King's College London | UK |
